# Strengthening health literacy research through consumer engagement: a qualitative analysis of building and maintaining partnership

**DOI:** 10.1093/heapro/daaf215

**Published:** 2025-12-19

**Authors:** Julie Ayre, Eliza Ferguson, Melody Taba, Olivia Mac, Kathleen McFadden, Marguerite Tracy, Geoffrey Edlund, Ivan C K Ma, Waren Nadesan, Julia Yan, Sharon Ng, Oliver Slewa, Kirsten J McCaffery, Danielle M Muscat

**Affiliations:** Sydney Health Literacy Lab, Sydney School of Public Health, Faculty of Medicine and Health, Edward Ford Building (A27), The University of Sydney, Sydney, 2006 New South Wales, Australia; Sydney Health Literacy Lab, Sydney School of Public Health, Faculty of Medicine and Health, Edward Ford Building (A27), The University of Sydney, Sydney, 2006 New South Wales, Australia; Sydney Health Literacy Lab, Sydney School of Public Health, Faculty of Medicine and Health, Edward Ford Building (A27), The University of Sydney, Sydney, 2006 New South Wales, Australia; Sydney Health Literacy Lab, Sydney School of Public Health, Faculty of Medicine and Health, Edward Ford Building (A27), The University of Sydney, Sydney, 2006 New South Wales, Australia; Sydney Health Literacy Lab, Sydney School of Public Health, Faculty of Medicine and Health, Edward Ford Building (A27), The University of Sydney, Sydney, 2006 New South Wales, Australia; Sydney Health Literacy Lab, Sydney School of Public Health, Faculty of Medicine and Health, Edward Ford Building (A27), The University of Sydney, Sydney, 2006 New South Wales, Australia; GP Clinical School, Faculty of Medicine and Health, Edward Ford Building (A27), The University of Sydney, Sydney, 2006 New South Wales, Australia; Co-SHeLL, Sydney Health Literacy Lab, Sydney School of Public Health, Faculty of Medicine and Health, Edward Ford Building, The University of Sydney, Sydney, 2006 New South Wales, Australia; Co-SHeLL, Sydney Health Literacy Lab, Sydney School of Public Health, Faculty of Medicine and Health, Edward Ford Building, The University of Sydney, Sydney, 2006 New South Wales, Australia; Co-SHeLL, Sydney Health Literacy Lab, Sydney School of Public Health, Faculty of Medicine and Health, Edward Ford Building, The University of Sydney, Sydney, 2006 New South Wales, Australia; Co-SHeLL, Sydney Health Literacy Lab, Sydney School of Public Health, Faculty of Medicine and Health, Edward Ford Building, The University of Sydney, Sydney, 2006 New South Wales, Australia; Co-SHeLL, Sydney Health Literacy Lab, Sydney School of Public Health, Faculty of Medicine and Health, Edward Ford Building, The University of Sydney, Sydney, 2006 New South Wales, Australia; Co-SHeLL, Sydney Health Literacy Lab, Sydney School of Public Health, Faculty of Medicine and Health, Edward Ford Building, The University of Sydney, Sydney, 2006 New South Wales, Australia; Sydney Health Literacy Lab, Sydney School of Public Health, Faculty of Medicine and Health, Edward Ford Building (A27), The University of Sydney, Sydney, 2006 New South Wales, Australia; Sydney Health Literacy Lab, Sydney School of Public Health, Faculty of Medicine and Health, Edward Ford Building (A27), The University of Sydney, Sydney, 2006 New South Wales, Australia

**Keywords:** health literacy, codesign, patient and public involvement/consumer involvement, qualitative methods, health education

## Abstract

Consumer engagement activities are variably implemented across research settings, and the ‘science’ of consumer engagement is still in its infancy. This study aimed to explore the experiences of community panel members who supported a university-based health literacy research group that delivers experimental and applied health research across a wide variety of health topics. An independent researcher interviewed participants, asking them to reflect on their understanding of health literacy research, their experiences on the panel, and strategies to improve the panel. De-identified transcripts were analysed using Framework analysis. Ten of the 13 Sydney Health Literacy Lab Co-SHeLL community panel members were interviewed (four men; seven born in Australia, six spoke English at home). We generated four themes from the data. The first three emphasized foundational elements that supported deeper consumer engagement: diversity and belonging, trust and respect, and effective structures. The fourth theme described how the panel provided mutual benefits to researchers and consumers through increasing skills and capacities, and that this improved the quality of the research. Participants described this as a gradual process that progressed over the course of the year. This rigorous qualitative evaluation identified values that may be important for long-term partnerships with consumers, particularly for health research groups with a broad remit (e.g. health communication and health promotion) that does not focus on a single health condition. Further research could evaluate this kind of consumer engagement activity over the longer term.

## Graphical Abstract

**Graphical Abstract:**
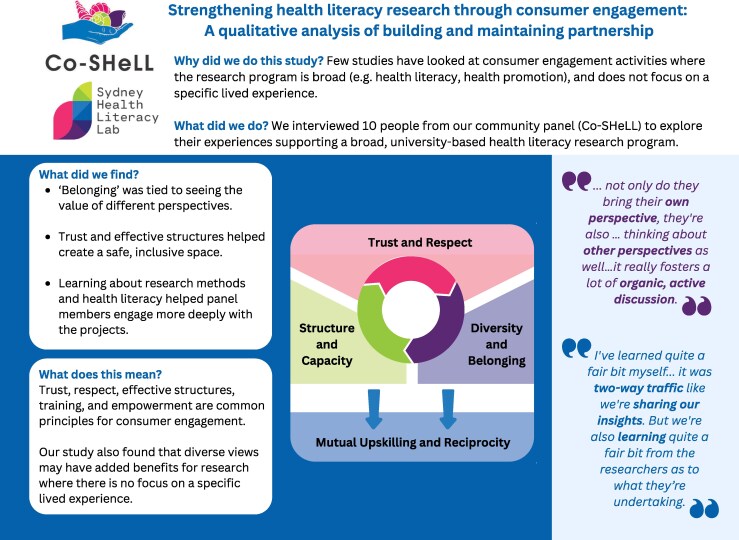

Contribution to Health Promotion

- It can be difficult for consumers to feel connected to research when it’s not directly tied to their specific lived experience, as is often the case for health promotion research.
- We explored experiences of community panel members who supported a broad health literacy research programme.
- Panel members felt a stronger sense of belonging when they saw how diverse viewpoints made for more fruitful discussions.
- Trust between researchers and panel members developed over time. Improvements to structures helped foster a safe environment.
- A better understanding of research methods, health literacy, and the research programme enabled panel members to engage more deeply.

Consumer engagement in health research encompasses a range of activities that seek to involve end-users in the research process, including patients and their carers, community members, and consumer representatives (Harrison *et al*. 2019). There are strong and long-standing ethical arguments for involving the people who are most likely to be impacted in the design and implementation of research (Thompson *et al*. 2009, Shimmin *et al*. 2017, Banner *et al*. 2019). There is also now increasing recognition that consumers bring valuable expertise to a project and can improve both the quality of research methods and the effectiveness of interventions (National Health and Medical Research Council 2024, National Institute for Health and Care Research 2024). As such, meaningful consumer engagement has become a growing priority for health research groups.

Several reviews have identified common elements that are thought to be important for successful consumer engagement. These are often discussed in terms of activities, principles, and outcomes. For example, Harrison *et al.* (2019) identified the four most common activities across 55 consumer engagement frameworks as (i) training, (ii) bidirectional communication, (iii) reimbursement, and (iv) selection of consumers based on skills and interests. The same study highlighted three fundamental principles: respect and equity, trust, and empowerment. Hoekstra *et al.* (2020) describe 15 anticipated outcomes of effective consumer engagement, ranging from individual researchers and consumers developing stronger skills and capacities through to delivering more relevant and effective research. Peters *et al.*’s (2024) recent review discusses these more immediate outcomes within the broader health context, describing further potential impacts on healthcare systems and community health.

Ideally, research teams should be aiming to build long-term partnerships with consumers. There are several reasons for this. Trust takes time to develop and strengthen and must be personally established with each new consumer who joins the team. Ongoing collaboration also creates unique opportunities for mutual learning and reflection, leading to a better understanding of each other’s goals, needs, and how to work together effectively. These points were highlighted by an evaluation of a consumer advisory group that supported cancer research over the course of 9 years (Milley *et al*. 2021). Consumers in this advisory group also described that a motivation for long-term involvement was their desire to improve outcomes and experiences for new cancer patients.

Systematic reviews show that most consumer engagement activities focus on a specific lived experience or clinical area (Wiles *et al*. 2022). Perhaps it is not surprising, then, that there is relatively little guidance about long-term partnerships for research groups that navigate diverse health topics rather than a specific lived experience. For example, research groups that focus on behavioural science, health communication, health literacy, and implementation science often apply their expertise across a range of health domains. This presents both challenges and opportunities: with such a broad (and sometimes non-clinical) remit, it can be difficult to find community members who feel personally connected across the range of projects. Equally, efforts to bring lived experience from diverse consumers into these projects stands to vastly improve their relevance and generalizability, and it is likely that insights from one project will be broadly applicable to others within the same research programme.

To address this gap in the literature, this study aimed to explore learnings and experiences of a consumer panel that had supported a university-based health literacy research group across a diverse range of clinical and non-clinical topics and research projects.

## METHODS

### Study design

This is a qualitative interview study. Ethical approval was obtained through the University of Sydney Human Research Ethics Committee (2024/HE000267).

### Sydney Health Literacy Lab and Community panel

#### Stated purpose and goal

Sydney Health Literacy Lab (SHeLL) is a university research group that conducts research spanning qualitative, quantitative, and participatory methods. Projects range from online experimental studies on health communication and behaviour through to applied health services research and implementation science. Projects relate to personal health literacy skills and knowledge, as well as the health information environment (e.g. use of jargon in health information and misinformation on social media). Many of the Lab’s projects relate to clinical groups and partner with consumers who have lived experience of a specific health condition, e.g. chronic kidney disease and type 2 diabetes. However, a substantial number of projects are also relevant to a general audience. The goal of the community panel, Co-SHeLL, was to create a safe and relaxed environment for two-way conversations, and in which community members felt supported to contribute to the Lab’s health literacy research projects, and to engage in consumer-led research.

#### Size and composition

Co-SHeLL was established in 2023 with seven members. In 2024, i.e. Year 2, the panel was expanded to 13 members, of whom four were returning members. Panel members were recruited into Co-SHeLL through the Lab’s existing networks and social media (e.g. via a flyer or social media post). People were eligible to join if they were adults living in Australia, were comfortable having discussions in English, and were able to use Zoom video conferencing to take part in meetings.

#### Consumer engagement principles

All activities were underpinned by the consumer engagement principles outlined by Harrison *et al.* (2019), i.e. respect and equity, trust, and empowerment. Our approach was also informed by the Agency for Clinical Innovation’s codesign toolkit (Agency for Clinical Innovation 2025). The toolkit provides practical guidance about ways of working that can foster four capabilities: collaboration, openness, respect, and empowerment (Box 1). Examples of how we sought to foster these capabilities are shown in Box 1.

Box 1. Consumer engagement capabilities*

**Table:** 

Capabilities	Examples of relevant actions and approaches
Respect	Cultivate an understanding, empathetic, and nonjudgmental culture Reminding panel members that all lived experiences, ideas, and opinions are valued and that everyone contributes expertise to the project At minimum, participants are reimbursed for their time and acknowledged in academic publications
Collaboration	Avoiding health and research jargon where possible Discussing that it is ok to disagree and that it is important to hear different sides of an issue Encouraging panel members to take turns to speak Creating a safe space, e.g. by emphasizing that discussions are confidential and that you can choose what you would like to share Providing different ways to communicate, e.g. verbally or through the chat function, through interactive online boards Demonstrating how feedback has shaped a project
Openness	Position the research team as ‘beginners’ or ‘learners’ and emphasizing to the panel members that everyone is learning, including the research team Setting the expectation that we will improve our processes over time Encouraging candid feedback Being curious and asking people to ‘say more’ on a topic rather than making assumptions about why they gave an answer Transparency, particularly around the reasons for decisions
Empowerment	Recognizing power imbalances and seeking to shift these imbalances where possible Providing support and flexibility where needed, e.g. around issues with digital technology, scheduling, and communication Facilitating discussions about future shared goals Emphasizing that one of the goals is to have greater consumer engagement further up the research pipeline Providing opportunities for training in research methods and topic area (health literacy)

*Adapted from Co-design toolkit (Agency for Clinical Innovation 2025).

#### Panel structure and activities

**Figure 1.**
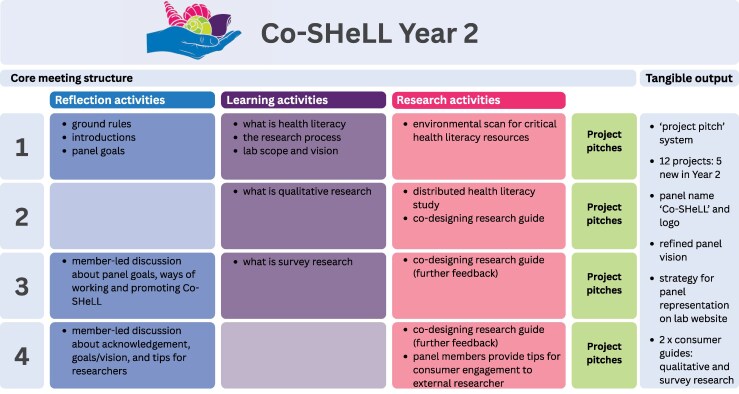
Summary of Co-SHeLL core meeting structure and outputs.

In both years (2023 and 2024), the community panel met online via Zoom four times for 2 hours in the evening (Year 2 topics shown in Fig. 1). The composition of the four ‘core’ meetings shifted over time. At the start, there was a greater emphasis on ensuring a strong foundational knowledge of health literacy and opportunities to work together on specific research projects through designated workshops. Each core meeting ended with ‘project pitches’ in which individual researchers could invite panel members to join their project outside of core meetings. Over time the core meetings then shifted to focus more on consumer-led and reflective activities with a view to enhancing the Lab’s consumer engagement efforts. In line with Health Consumer NSW reimbursement rates, panel members received an AUD$50 gift card for each hour of their time to acknowledge their contribution. In between meetings, panel members could hear about the Lab’s news via the quarterly newsletter as well as posts on social media (Facebook, Instagram, and LinkedIn).

### Participants, recruitment and data collection

Panel members were invited to take part in evaluation interviews after the final meeting in Year 2 (November 2024). To foster open and honest discussions, participants were informed that they would be interviewed by a researcher who had not been involved in the panel (Author 2) and who had no existing relationship with the panel members. After providing informed consent, participants completed demographic and health experience questions, including the single item health literacy screener (Wallace *et al*. 2006).

Author 2 carried out semistructured interviews with participants that asked about their understanding of health literacy research, their experiences of being involved in the panel, and strategies to improve the panel (Supplementary A). Participants were interviewed between 22 November 2024 and 16 January 2025. Interviews lasted 40 minutes on average (range: 23–53 minutes). Author 2 noted impressions and reflections after each interview.

### Analysis

Quantitative data (e.g. demographics and attendance at meetings) were analysed descriptively using Microsoft Excel (version 16.94). Qualitative data were analysed thematically using Framework analysis (Gale *et al*. 2013). Author 2 familiarized herself with the data by reading transcripts and taking reflective notes. Codes were applied in a predominantly inductive manner and were not predefined. This was carried out by going through the data line by line and attaching a label to small sections of the text that captured ideas that the analyst interpreted as important or relevant. Authors 1 and 2 independently coded three interview transcripts. They discussed their codes and identified potential patterns or ‘categories’ of codes that could be used to develop a preliminary thematic framework. The preliminary framework was then discussed with study authors (seven Lab staff and six Co-SHeLL members) to further refine the themes. At this point, Author 2 then applied the thematic framework to more of the data using Microsoft Excel, organizing transcripts into a matrix of interview (rows) and themes (columns), meaning data could be compared by interview or theme.

This preliminary framework was gradually refined by going back to the data and applying codes to the framework, reflecting on the meaning of the themes and their relationship to the underlying data, and then refining the framework based on these reflections. The framework was refined, e.g. to ensure themes had sufficient depth and narrative quality, had clear boundaries, and were supported by the data. This was an iterative process that incorporated more of the dataset over time. During this process, themes and relationships between themes were described, mapped, and interpreted.

Authors applied a critical realist epistemology (Maxwell 2012), recognizing that a real world exists independently of our perceptions and that the way we come to understand and make sense of the world is subjective. As such, our analysis sought to provide an interpretation of reality rather than determine facts or truths. The lead analyst (Author 2) had training in psychology, and by virtue of working within a health literacy research group that was situated within a public health discipline, she places value on inclusive communication and improving equity in research processes. More specifically, her research experience had focused on person-centred health topics, e.g. shared decision-making, health literacy, and the lived experiences of carers. She had limited personal experience in consumer engagement activities. As someone who had not previously been involved in the project and who had never met the participants, we hope that this encouraged a more open and candid dialogue during the interviews.

## RESULTS

Twelve of the 13 community panel members consented to take part. Ten were able to find time to take part in an interview. Participant characteristics are displayed in Table 1 and Supplementary Table S1. Almost all participants who consented had attended the four core meetings in Year 2 (*n* = 11). Participants had contributed to a total 12 Lab projects that were initiated or active in Year 2. Involvement activities included grant preparation, developing the research question, consultation, codesign workshops, data collection and analysis, and contributions to academic manuscripts. Six of the 10 participants identified as women, nine were aged between 20 and 49, and three lived outside of a major city.

**Table 1.** Participant characteristics.

Characteristic	*N*
Age	
20–29	3
30–39	3
40–49	3
50–59	0
60+	1
Gender identity	
Man	4
Woman	6
Residential location	
Major cities of Australia	7
Regional	3
Country of birth	
Australia	7
Other	3
Health literacy	
Limited/marginal	1
Adequate	9
Language spoken at home	
English	6
Other	4
Education	
Less than undergraduate degree	1
Undergraduate degree or above	9
Long-standing health condition	
None	3
One	3
Two	3
Three or more	1
Attendance at core meetings	
Four meetings (all)	9
Three meetings	1
Total	10

**Figure 2.**
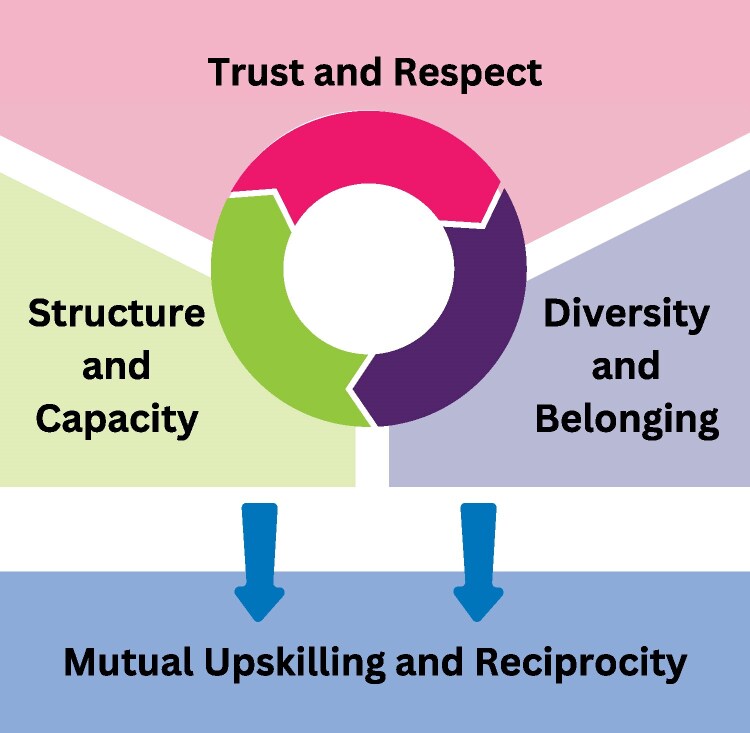
Depiction of themes developed from the qualitative evaluation of the Co-SHeLL community panel.

We developed four themes to describe participants’ experiences of the community panel (Fig. 2). The first three describe foundational elements (belonging, trust/respect, and structure) that helped create an open supportive environment in which panel members described feeling safe to share their stories and contribute to discussions. The fourth theme describes how these elements allowed researchers and panel members to experience two-way benefits from the Co-SHeLL consumer engagement activities.

### Theme 1: Diversity and belonging—‘They bring in their own…perspective, [and] they’re also thinking about other perspectives as well’

This theme highlighted the importance of representing diverse community voices and ensuring that consumers from a range of backgrounds and lived experiences were able to access and engage with the community panel meetings and research projects. Participants highlighted that collectively the panel members were able to speak from a variety of experiences relating to health and the healthcare system, professional expertise, experiences of being part of a culturally and linguistically diverse community, living rurally, being member of the lesbian, gay, bisexual, transgender, intersex, queer, asexual and other sexually or gender diverse (LGBTQIA+) community, and living with disability. They discussed how, as a group, they could better bridge the gap between communities and health research and increase the nuance and fruitfulness of discussions about consumer experiences, even though they were not connected through a shared experience of a specific health condition:

*I really like the diversity of that. I feel like… everyone’s very active, and that really allows for, like a more fruitful conversation and discussion, and I love that everyone brings their own kind of like perspective, because I think everyone’s from like different way of life. I mean, like different cultures, different ethnicities, etc. …not only do they only bring in their own perspective, they’re also … thinking about other perspectives as well…Yeah, so like, I think it really fosters a lot of like organic active discussion.* (P02; W, 20–29 years)

Another participant reflected on her advice for consumers looking to join research projects and shared that community members might feel as though their personal experiences aren’t what a research team is looking for, particularly if they didn’t have a PhD:

*A lot of the time people hesitate because they're concerned that I might not be the right person, or I might not have the background knowledge with these sorts of things…* (P07; W, 40–49 years)

This participant went on to explain that over the course of the panel, they better understood why their experiences mattered and strengthened their belief in the value of diverse perspectives from people without extensive research or medical training. In this way, the diversity of the panel helped her develop a sense of belonging and the value she brought to discussions:

*That’s the first thing to consider, is just because you might not understand everything [about health or research], doesn't mean that you shouldn't do it.* (P07; W, 40–49 years)

Lastly, whilst participants reported positively on the diversity of the panel, they also discussed a range of strategies to better support diverse communication needs, e.g. using the breakout room feature in Zoom so that the groups were smaller, opportunities for asynchronous feedback, and deliberately pausing before starting discussions:

*…Some people are really fast at thinking things up. I’m not one of those people. I take time to process things*… (P08; W, 60+ years)

Some participants were also interested in having a larger panel so that there was capacity for specific subgroup panel discussions:

*I want them to grow it …have a youth consumer panel, have a First Nations consumer panel, have a seniors consumer panel. Have a CALD representatives consumer panel like I can keep going on….* (P01; M, 30–39 years)

However, they also acknowledged that this larger panel may feel less personal and might risk the strong sense of belonging they had developed:

*I mean it’ll always be good to have an even larger panel… But then I guess the challenge with that is trying to incorporate everyone … especially if it's virtual …it’s probably gonna be a bit harder* (P10; M, 30–39 years)

### Theme 2: Trust and respect—‘I felt like we’re actually being listened to here’

This theme underscored the importance of building trust between researchers and community members and amongst community members themselves. Trust was developed over time, and participants appreciated the intentional way that the researchers created a ‘friendly and super welcoming’ environment and ‘very safe space’ to share their views:

*In the past I think sometimes it’s been, “Hey, this is what we’re going to tell patients they need to do”. But now it’s like, “Hey… this is what we’re going to work with our patients to involve them in doing”* …*Everybody [in the lab] seems to be very much about involving… consumers in decision making* (P07; W, 40–49 years)

Participants described that an important way of fostering trusting and respectful relationships was to address the traditional power imbalance between researchers and community members:

*[the lab is]…kind of like closing the distance between researchers and consumers… because, I guess, for people outside academia those professors always sound very unreachable…*  *[Within the lab] there’s a very, I don’t know, friendly vibe, and then you feel very comfortable… I’ve never heard [researcher] say I have a PhD, you have to listen to me…I don’t hear that from the Lab, which is very nice.* (P05; M, 20–29 years)

Participants described how researchers could address this power imbalance by showing that they appreciated feedback and active discussions and by reducing feelings of being ‘just there as a token mechanism’. Participants gave examples of how researchers demonstrated these attributes by addressing as many recommendations as possible and actively communicating how consumer input had shaped the research:

*They’re very proactive. They take on suggestions. If they get 50 suggestions, 49 of those are implemented, or at least acknowledged…I really appreciate that our time is valued.* (P01; M, 30–39 years)

Updates on study progress also contributed to a sense of trust. Some contrasted this with negative experiences where they ‘[didn’t] hear about [the project] at all’ following their initial involvement in the project, and they ‘don’t know what’s happened to it’. Updates were particularly important when meetings were intermittent:

*She keeps me up to date on…how it’s progressing, what’s going to happen, etc. So like, I’ve really had a great time experience with it…I think that’s quite important, because sometimes you don’t know what the next steps are or there’s a long time between each of the sessions…*  *So it’s good to know… that that they haven’t forgotten about it, like it’s still ongoing. It’s just taking some time.* (P02; W, 20–29 years)

Lastly, some participants highlighted that more formal aspects of recognition were important for feeling valued. Participants often desired formal recognition including remuneration. They also described other forms of formal acknowledgement such as notices in a bulletin or newsletter. A few hoped for recognition through coauthorship on papers and grants in the future:

*… maybe they’ve contributed a few lines [to a manuscript]…having that chance, perhaps to contribute to the … the actual writing process.* (P06; W, 30–39 years)

One participant believed that the current form of compensation for the community panel, gift vouchers, was ‘a bit tacky’ and suggested an honorarium as a better form of remuneration whilst also acknowledging the complexity of this arrangement in the context of ‘the administrative and the financial parts of universities’:

*So if you know genuine about doing the long term venture in this, maybe think about things like an honorarium…You know, pay people for this… Let’s structure that role and actually go, okay, well, we’re engaging you as a representative for 12 months. I think it’s, it feels like it’s time for that.* (P03; M, 40–49 years)

### Theme 3: Structure and capacity—‘I feel like it’s time to streamline how it happens’

This theme examined factors related to the structure and format of the community panel and its meetings and how these influenced participants’ experiences. Generally, participants felt positively about the meetings, but would prefer them to be more frequent:

*Maybe the meeting frequency can be different, because right now I think we don’t meet very often. and that’s just hard to keep track of what happened since the last meeting…*  *I guess another type of communication or newsletter will be nice just to keep them in the loop.* (P05; M, 20–29 years)

Participants described how this would make it easier to remember activities from the previous meeting, increase a sense of cohesiveness within and across projects, and slow the pace of each session to allow for more discussion time:

*[in] 15 minutes or 20 minutes …it’s hard to convey really what [the researcher is] talking about. So if we can …listen to their presentation [in a longer format]… you’d have a better understanding of their research, because you only having some snapshots here and there*. (P04; W, 40–49 years)

In general, there was a sense that some formats worked better than others and that they preferred consistent meeting structures because this helped them know what to expect. For example, some participants enjoyed the ‘project pitch’ activity, in which researchers would briefly talk about their research project and connect with interested consumers in a separate meeting:

*I like the fact that it’s short, sharp bites to not too long, because some of us…if we had to sit for two hours and listen to some research that we weren’t interested in, I think that that would be too much. So that short, sharp bites. And then it’s like, if you’re interested, you can contact this person* (P06; W, 30–39 years)

Participants also described a preference for presentation styles that used common, everyday language and reported that a few presenters had made assumptions about their knowledge of research and medical jargon. This made it more challenging to engage in the discussions:

*So [some of the researchers] they don’t really realize … [that] the panel that they’re talking to like don’t actually know much about it. So I think they sometimes can go like into really deep detail, or go on and on.* (P02; W, 20–29 years)

### Theme 4: Reciprocity and mutual upskilling—‘It’s two-way traffic’

This theme discusses the genuine reciprocity and mutual benefit that can be experienced by both community panel members and researchers when community voices are actively sought out and amplified in the research process. Every participant spoke positively about the learning experiences they had gained through their participation on the panel.

*I was pleasantly surprised that … because I could get involved in various studies about different things. And I’ve learned quite a fair bit myself, you know, like it was two-way traffic like we’re sharing our insights. But we’re also learning quite a fair bit from the researchers as to what they’re undertaking. So that was fantastic.* (P04; W, 40–49 years)

Most panel members believed their understanding of health literacy had improved. This frequently included a broadening of their mental model of health literacy to include community and systemic factors, as well as factors at the individual level.

*…you engage with the whole, not only community, but a whole series of practitioners through that health journey from your GP, to specialists, to the organizations…[health literacy], it’s public, it’s the media, and that it’s not as simple as, you know, the doctor tells you X.* (P03; M, 40–49 years)

About half of the participants discussed how their understanding of research processes had improved. This included a better understanding of project timelines:

*I love that they do discuss it in detail. So it’s given me like a deeper understanding of what goes on in the background. Because a lot of these research projects, they like span for years right? But like you don’t really know why it spans for years so like this has just made me understand it better…* (P02; W, 20–29 years)

Some participants felt that the knowledge and experiences gained on the panel had helped them become more deeply engaged in the research. This was demonstrated through several mechanisms. Firstly, through reports of feeling more comfortable challenging each other and members of the research team:

*…what’s been particularly good about the last meeting or two is the debate. At least five or six of us are quite comfortable now, saying I take your point, but I don’t agree with that. At one point we were just sitting watching slides click past on our computer, [and this has changed now] to understanding a bit about each other's background and actually challenging each other in a general kind of professional way. That that felt really good…* (P03; M, 40–49 years)

*But over time I think I’ve really been able to provide my feedback…and… be able to … question, and sometimes challenge the researchers* (P02; W, 20–29 years)

Deeper engagement was also demonstrated through participants’ desire to better understand and influence the Lab’s research agenda. One participant described the goal of ‘a model where it [the community panel] is like 50% community driven, 50% researcher driven’ (P03; M, 40–49 years). To achieve this goal, this participant talked at length about the importance of understanding ‘Where have we been, and…where could we go? … If you look at that map, you find there’s big areas where you’re not engaging the community yet, and maybe you could’.

Beyond engagement in the panel, a few members hoped that it might support them to develop specific research skills and knowledge that they could then go on to share with their community:

*Maybe in 5 years’ time we might be a bit more knowledgeable and confident enough to do that [contributing to writing journal manuscripts] and you know, familiar with the research process and how universities work and things… perhaps you could have a presenter who could even teach us a few things about the writing process, or, or how medical online journals work* (P06; W, 30–39 years)

*… I am really interested in things happening for my community…*  *and just the ability to potentially bring back some of those ideas and share them with, with some of the community groups that I’m involved in here locally and say, Look, this is the way that somebody else was doing this study or this group to share this bit of knowledge. Is that a way that we could apply in this setting?* (P07; W, 40–49 years)

Lastly, participants also described how researchers and the Lab overall had benefitted from the involvement of community members. Many panel members noted that including consumer voices and perspectives along the research pipeline ensured that research addressed unmet community needs, and was relevant, acceptable, and accessible to the community:

*The more I got involved, the more I saw the, the usefulness of, you know, getting the perspective from the community as well, because I’ve been involved in a few of those studies throughout the year, and you could see that sometimes they had their research hat on without really understanding the community's perspective. So, it was good that there was that sharing of insights and information between both parties. So yeah, I was, yeah, I was really interested in that sense to be able to participate on that level* (P04; W, 40–49 years)

One panel member also observed an improvement in the researchers’ skills and approach to facilitating community involvement in research:

*I think when we started it was more about this, “we’re about to launch something” [consumer involvement later in the research pipeline]. And then, as the panel’s gone on, it’s gone earlier and earlier in the process. And now we’re [being asked] “what do you think about this idea or pitch?” And now we’re even going, okay, “well, we did that journal article. We got that grant. We launched this tool. What was the feedback evaluation monitoring? How could we improve?” So, it it’s gone from being very focused on the kind of beta test, to, okay, let’s actually be a participant in the process. And, and that’s I think that’s really beneficial…I do feel like the lab’s learned a lot and vice versa.* (P03; M, 40–49 years)

## DISCUSSION

This study explored the experiences of consumers who supported a variety of health literacy research projects for a university-based research team over a 12-month period. Findings emphasized how diversity and belonging, trust and respect, and effective structures were fundamental to an open and collaborative partnership. Consumers recognized that their involvement brought personal benefits (e.g. greater understanding of health literacy and research), as well as perceived benefits to the projects (e.g. more impactful consumer engagement), and to individual researchers (e.g. improved facilitation skills and consumer engagement strategies).

The findings are largely consistent with a growing body of research that highlights key concepts for successful consumer engagement, including Harrison et al.’s three fundamental principles of respect and equity, trust (through transparency and honesty), and empowerment (e.g. through shared decision-making and ownership) (Harrison *et al*. 2019, Hoekstra *et al*. 2020, Peters *et al*. 2024). Similarly, the benefits reported by participants in this study reflect the types of benefits that are typically identified in consumer engagement evaluations, e.g. more relevant and higher quality research, stronger relationships between consumers and researchers, and improved knowledge and skills (Hoekstra *et al*. 2020, Peters *et al*. 2024). However, Peters *et al.* (2024) also described potential impacts that extend beyond the codesign group (e.g. changes to the healthcare system and community health). These concepts did not feature strongly in our findings and may reflect the broad scope of the projects that consumers were involved in (e.g. tips for using social media for health information) and which did not include research on the delivery of specific health services within a clinic or hospital.

Diverse representation is also an important concept, particularly for consumer engagement activities with a focus on inclusive and equitable outputs. However, Harrison *et al.* (2019) reported that diverse representation was only identified in 22% of frameworks; nor is it a concept that is typically included as an explicit evaluative outcome (Peters *et al*. 2024). Diverse consumer representation is a well-documented and persistent challenge (Synnot *et al*. 2022, Brammall *et al*. 2024) that is even reported by consumers themselves (Ayre *et al*. 2023, Synnot *et al*. 2024). The pursuit of more diverse and intersectional representation may also benefit from a shift from expecting that a consumer’s perspective should be representative of their community, towards the idea that representativeness is only achievable through wide inclusion (Scholz *et al*. 2024). Scholz *et al*. (2024) argue that this shift can help reduce power imbalances because it removes the question about whether a consumer is ‘representative enough’ and reduces the risk of silencing their expertise. Our findings suggest that participants in our study may have experienced this conceptual shift themselves and provide evidence that perceptions of diversity may have also helped to reinforce that all experiences matter, as Scholz *et al*. (2024) suggested. In the context of health literacy and health promotion, efforts to help consumers explore their understanding of consumer representation are likely to be particularly important for those consumers who do not have an established professional role as a consumer representative and for people without formal medical or research training. We also recognize that diversity amongst the Lab’s consumer panel can be further improved. We have addressed this in our recruitment and communication strategies for the next phase of Co-SHeLL.

Another key finding was that although we had identified codesign principles and capabilities at the start of the project, participants felt that consumer engagement processes improved over time. This study identified several processes that may have influenced this perception. For example, participants described how they became more trusting and more deeply engaged in the projects as they saw how their feedback shaped the projects and as they became more comfortable to discuss their feedback openly with each other and the research team. They also described how over time, they felt the researchers were trying to partner with consumers earlier in the research pipeline and that they felt more confident to deeply engage with the research projects as they developed a stronger shared understanding of health literacy concepts and research.

The findings from this study are consistent with Greenhalgh *et al.*’s (2019) advice that building a ‘tailored’ consumer engagement framework may be more successful than using those of other research groups in an off-the-shelf fashion and adds that a tailored framework can be refined over time through evaluation and feedback. This flexible approach to consumer engagement may be particularly important in cases such as ours, where the goals of the panel were intentionally broad and focused on long-term goals and where members supported a programme of research that is not focused on a specific lived experience. For example, in this project, it took time for consumers to understand the ‘bigger picture’ of the Lab’s research programme beyond any individual project, including different kinds of study methods and consumer engagement activities. This has provided us (both researchers and the consumers) with valuable insights to develop our own repertoire of consumer engagement activities and appropriate supports and guidance for these activities.

This study had several strengths. Participants were interviewed by an independent researcher who had not been involved in Co-SHeLL or any related projects. A number of panel members were involved in the analysis, ensuring that we centred consumer perspectives in our findings. The key limitation was that we were unable to recruit and interview all panel members. This may relate to the timing of recruitment and other personal priorities. It is unlikely to reflect a lack of engagement or positive sentiment towards the panel, given that the people who we were unable to interview had full attendance and core meetings. Findings are likely to be specific to university-based research teams undertaking projects across a variety of lived experiences (or for which panel members do not necessarily share a common health condition). The findings also reflect experiences over the course of a year, in most cases. We would expect these experiences to change over time as Co-SHeLL members and researchers develop deeper connections.

## CONCLUSION

This study presents a rigorous evaluation of a consumer panel that supports a single university-based research team focused on health literacy research across a variety of health topics, over an extended period. For research groups looking to establish long-term partnerships, our findings emphasized the importance of trust and respect, diverse representation, and effective structures. Together, these formed a strong foundation through which panel members and researchers could improve their knowledge and skills for effective consumer engagement activities. Further research could explore the role of potential consumer engagement strategies identified in this study (e.g. more frequent meetings, use of social media, and research training) and also evaluate this kind of consumer engagement activity over the longer term.

## Supplementary Material

daaf215_Supplementary_Data

## Data Availability

The data underlying this article will be shared on reasonable request to the corresponding author.
