# Supplementary material for: Strengthening health literacy research through consumer engagement: a qualitative analysis of building and maintaining partnership
Source: Health Promot Int. 2025 Dec 19;40(6):daaf215. doi: 10.1093/heapro/daaf215 (PMC12715179; doi:10.1093/heapro/daaf215)
Supplement: daaf215_Supplementary_Data [file daaf215_supplementary_data.docx]

# Supplement A: Interview topic guide

1. What does health literacy mean to you? What perspective do you feel you bring to health literacy research? Has this changed over time?
2. What expectations did you have when you joined the health literacy research group?
3. What were your general impressions of the consumer panel? prompt for positive/negative (time, organisation, composition and membership, activities)
4. What was your experience like being involved in the lab’s health literacy projects. Prompt for e.g. What kinds of tasks or activities worked well? Not so well?
5. What are the main things you learned about health literacy, research and consumer involvement – have these changed over time?
6. What directions would you like the research panel to go in over the next year? Five years?
7. Do you have any advice to community members interested in being involved in research as a consumer representative?

# Supplement B: Tables

**Table S1. Characteristics of participants who consented to take part in the study***

| **Characteristic** | **n** |
| --- | --- |
| **Age** |  |
| 20-29 | 4 |
| 30-39 | 4 |
| 40-49 | 4 |
| 50-59 | 0 |
| 60+ | 1 |
| **Gender identity** |  |
| Man | 4 |
| Woman | 9 |
| **Residential location** |  |
| Major Cities of Australia | 10 |
| Regional | 3 |
| **Country of birth** |  |
| Australia | 9 |
| Other | 3 |
| **Health literacy** |  |
| Limited/marginal | 1 |
| Adequate | 11 |
| **Language spoken at home** |  |
| English | 8 |
| Other | 4 |
| **Education** |  |
| Less than undergraduate degree | 1 |
| Undergraduate degree or above | 11 |
| **Long-standing health condition** |  |
| None | 3 |
| One | 3 |
| Two | 4 |
| Three or more | 2 |
| **Attendance at core meetings** |  |
| Four meetings (all) | 11 |
| Three meetings | 1 |
| **Total** | **12** |

*Includes those who consented to take part in the study. Two of these people did not take part in an interview.
